# Human Recognition: The Utilization of Face, Voice, Name and Interactions—An Extended Editorial

**DOI:** 10.3390/brainsci14040345

**Published:** 2024-03-30

**Authors:** Guido Gainotti

**Affiliations:** Institute of Neurology, Università Cattolica del Sacro Cuore, Fondazione Policlinico A. Gemelli, Istituto di Ricovero e Cura a Carattere Scientifico, 00168 Rome, Italy; guido.gainotti@unicatt.it; Tel.: +39-06-30156435

The many stimulating contributions to this Special Issue of *Brain Science* focused on some basic issues of particular interest in current research, with emphasis on human recognition using faces, voices, and names. I therefore thought that it could be useful to group these papers, after a short introduction, on the basis of the principal problem that they consider.

## 1. Introduction

Many of the earliest investigations into the neural substrates underlying human recognition using the face, voice, and name considered the basic problems of this research domain. In particular, emphasis was placed on the brain structures underlying these cognitive skills, the selectivity of work performed by these structures, and the main similarities and differences between these recognition modalities. These studies originally focused attention on the brain structures involved in facial recognition, showing that face-sensitive areas are located in the fusiform face area (FFA) [1,2] and the occipital face area (OFA) [3,4]. They also suggested that the occipital face area could be principally involved in the processing of visual sensory information. Conversely, they thought that the fusiform face area might be primarily implicated in facial identity recognition [4,5,6]. Similar observations were made when investigating the brain areas involved in voice recognition using the voice-sensitive regions found in the superior temporal gyrus/sulcus (STG/STS) [7]. In analogy with the distinction between the functions of the OFA and of the FFA, it was suggested that posterior STG/STS might be more involved in the processing of acoustic properties of voices, with the anterior STG/STS potentially thought to be more related to voice-based identity recognition [8,9,10]. A further similarity observed between the facial and vocal components of human recognition systems was that, even if face- and voice-selective areas were present in both hemispheres, the right-sided areas were more important for both facial [6] and vocal processing [11]. The right lateralization of these (non-verbal) human recognition modalities contrasted with the left lateralization of the (verbal) name recognition modality. This observation was consistent with the results of clinical studies carried out in patients with unilateral brain lesions [12,13] and of experimental investigations conducted in healthy subjects [14]. A final similarity between facial and vocal recognition modalities was that that, in addition to these core networks of components, both these human recognition modalities also comprise extended networks, permitting techniques to advance from unimodal processing into multimodal person representations. In contrast with these similarities, which inspired Belin et al. [8] and Yovel and Belin [15] to advance the notion that voice may be considered an ‘auditory face’, other authors have stressed the existence of important differences between these two modalities of human identification. Neurobiological investigations have, for instance, documented the lower degree of functional specificity shown by areas involved in voice perception in comparison to those involved in face perception [16,17]. The greater difficulty documented by several authors (e.g., Refs. [18,19,20]) in vocal rather than facial human recognition is probably related to this difference. Another source of dissimilarity stems from the different temporal dimension of facial and vocal processing: faces can be discerned from a single snapshot [21], whereas voices constitute dynamic stimuli for which receivers must integrate information over time. Other investigations concerning facial, but not vocal, recognition modalities have highlighted two problems: (a) the face inversion effect, namely the observation that turning a picture of a face upside-down makes its identification much more difficult than the recognition of other objects [3,6]; and (b) the question of which relations between the pathways allow for, respectively, facial identification and the recognition of facial emotional expressions. The classical views on this subject assumed that two distinct pathways could allow for the recognition of facial identity and of emotional facial expressions. This was based on the assumption that the encoding of the structural aspects of facial identity should be mainly performed via the ventral visual pathway, whereas the view was held that the processing of facial expressions should be performed using the dorsal visual pathway [22,23,24,25]. However, Duchaine and Yovel [6] have proposed a revised framework in which OFA and FFA are engaged in processing facial shape, contributing to both facial identity and facial expression recognition. Furthermore, more recent animal studies have revealed the presence of information about facial identity and facial expression in the middle dorsal face area of macaque monkeys [26].

In more recent times, the attention of researchers has shifted from the basic components of human recognition systems to more complex functions. These investigations have mainly concerned the interactions that could exist between perceptual channels, which process faces and voices before the level of the ‘person identity nodes’ (PINs). The latter, according to cognitive models of recognizing familiar people (e.g., Refs. [27,28]), should allow for the identification of a person characterized by a given face and voice, giving access to the corresponding semantic (biographical) information. The earliest data to raise this problem were obtained by Schweinberger et al. [29,30], who observed that famous faces, but not famous names, caused a long-term repetition priming effect in the recognition of famous voices. Schweinberger et al. [29] concluded that these findings might be related to “perceptual links” between faces and voices, and this suggestion was strongly supported by data obtained by following authors. For instance, von Kriegstein et al. [31,32] measured, the brain activity of the FFA during identification tasks by means of functional magnetic resonance imaging (fMRI). These tasks saw subjects focus on either the speaker’s voice or the verbal content of sentences. These authors showed that familiar persons’ voices activated the FFA when the identification task was to focus on the speaker’s identity. Further, they demonstrated that, in these conditions, a functional connectivity between FFA and STS was obtained during familiar speaker recognition. In more recent years, other studies have tried to identify the main factors underlying the formation of these connections. Some of these studies have proposed the “metamodal” principle of brain organization [33], which maintains that the similarities in the cognitive and neural mechanisms underlying facial and vocal perception may lead to the recognition of identities across auditory and visual modalities. Other authors (e.g., Refs. [29,30,31,32]) have suggested that an important factor leading to the formation of interactions between facial and vocal processing channels could be the familiarity of stimuli. There are two important factors supporting this suggestion. The first is that a ‘familiarity feeling’ is automatically generated when a perceptual pattern matches a stored representation of the same stimulus and that the generation of the corresponding ‘familiarity feeling’ is the first step of the recognition process. The second factor is that the production of a familiarity feeling for a given face or voice is a right-lateralized component of the process of human recognition. The brain structures involved in facial and vocal recognition are just as right-lateralized. Clinical studies that have investigated the lateralization of early and late components of facial (prosopagnosia) and vocal (phonagnosia) recognition disorders have also shown that the disruption of feelings of facial and vocal familiarity usually occurs due to right brain lesions, whereas the unsettlement of name-related familiarity feelings is due to left brain damage [34,35,36]. Even if all these investigations show that interactions exist between the perceptual channels processing faces and voices before the level of PINs, it remains possible that face–voice integrations may also be mediated by a later (post-perceptual) stage, as implied by classical models [27,28]. This proposal is, however, not supported by the results obtained by investigations that tried to evaluate the interactions between semantic information and facial or vocal learning.

Several aspects of these questions are taken into account in this Special Issue. They are considered here shortly. This is first performed by taking into account the problems relating to unimodal facial and vocal perceptual modalities and then the more general problems regarding the interactions between different perceptual channels. The importance of these connections in the functional reorganization of neural networks for human recognition in blind subjects and the role played by familiarity and by brain asymmetries in modality-specific and inter-modal interactions are also considered shortly.

## 2. Papers Specifically Dealing with the Brain Structures and Mechanisms Involved in Modality-Specific Aspects of Facial and Vocal Recognition

Since problems concerning facial processing have been more extensively studied than those relating to voice recognition, the number of papers dealing with facial recognition in our Special Issue surpasses that of articles concerning voice identification. Of the papers specifically considering face recognition, the contribution of Rossion et al. [37,38] is particularly interesting. This submission summarizes a 10-year research program that combined, in epileptic patients, the recording of intracerebral activity in the ventral occipito-temporal cortex, resulting in rapid periodic visual stimulation. Indeed, this study has increased our knowledge of the neural basis of face recognition, reconciling the wide distribution of neural face recognition activity with its (right) hemispheric and regional specialization and confirming the spatial dissociations in category selectivity between faces and other meaningful visual stimuli.

In another contribution, Hagen et al. [39] have tried to clarify the mechanisms underlying the ‘face inversion effect’ [3], evaluating the plasticity of the neural processes involved in this effect after extensive visual training in adulthood. At the behavioral level, the authors reported a significant reduction in the face inversion effect. This correlated with data obtained on a neural index of facial identity recognition, confirming the involvement of a substantial degree of plasticity in processes that are critical for facial identity recognition.

Two other important papers in this Special Issue factor in the problem of the relationship between the recognition of facial identity and the identification of emotional facial expressions. In particular, Schwartz et al. [40] have reconsidered the question of the neural pathways that enable the detection of facial identity and of facial expression, whereas Chauhan et al. [41] have tried to clarify if personal familiarity, which facilitates the rapid and optimized detection of faces, also promotes the detection of facial expressions. Schwartz et al. [40] have challenged the classical view, which assumes that facial identity and facial expression may be processed by segregated neural pathways [22,23,24,25], by testing the hypothesis that integrated representations of identity and expression arise spontaneously within deep neural networks. They used two different datasets to train a deep convolutional neural network in order to label face identity and facial expression, respectively, showing that information about identity and expression are encoded within common brain regions. The authors also showed, however, that features used to distinguish between identities and expressions become increasingly orthogonal from layer to layer, suggesting that deep neural networks can disentangle representational subspaces corresponding to facial identity and facial expression. On the other hand, Chauhan et al. [41] measured, in a visual search paradigm, accuracy and response time to two different emotional expressions (anger and happiness) displayed by familiar and unfamiliar faces in order to determine local facial features contribute to both face identity and facial expression recognition. These authors showed that personal familiarity facilitates the detection of some (angry) facial expressions, but not of others (happy), and assumed that the advantage of familiar face recognition may rely on a feature-based type of processing, supporting the claim of Kaufmann and Schweinberger [42] that there is a relationship between the recognition of familiar faces and the recognition of facial expressions.

Only one article in this Special Issue concerns aspects of voice-based identity recognition specifically. In this paper, Stevenage et al. [43] examines voice processing in challenging listening conditions, starting from the premise that voice-based identity processing depends on both the ability to tell two instances of different speakers apart and the capacity to identify two instances of the same speaker. Since previous research only examines these vocal processing capabilities under relatively common listening conditions, the authors conduct two experiments that employ challenging listening tasks, determining just how good their subjects are in terms of these voice processing tasks. The authors show that vocal identity processing is a highly adaptable task that is assisted by familiarity with the speaker, suggesting that stored mental representations may be viewed as regions that capture and reflect vocal variability within a speaker, and also that voice processing is far better than was previously presumed.

## 3. Articles Dealing with the Interactions between Different Channels of Human Recognition and their Possible Underlying Mechanisms

Various problems relating to the interactions between different channels of human recognition are taken into account, sometimes with apparent conflicting results, in this Special issue of *Brain Science*. The hypothesis that interactions between different channels of human recognition may not only occur before the level of the ‘PINs’, but also at a later (post-perceptual) stage, is tackled by Frannson et al. [44]. These authors try to evaluate whether perceiving a person’s face or voice enhances the encoding of his/her biographic data. They perform three experiments, in which subjects learn the biographic data of an individual, whether or not they are associated or not with vocal/facial perception. After learning, biographic data alone are presented to the same subjects who, in the test phase, are tested for both familiarity and their capacity to match biographic data to a name. The results show that the simultaneous processing of autobiographic and perceptual representations of humans does not modulate the encoding of biographic data, confirming that the interactions between autobiographic information and channels of human recognition do not occur after the level of the ‘PINs’. The same research group [45], however, also present results that are at least partly inconsistent with models indicating that interactions between different channels of human recognition can occur before the level of the PINs. These authors show, in fact, that subjects who observe faces associated with vocal data, biographic data, or both, do not perform better in the retrieval phase, on either familiarity tasks or face-to-name identification tasks, than those who undergo the encoding of faces without additional information. Since these results contrast with most data reported in the literature and in other contributions to this Special Issue, I think that these inconsistencies could be due to two methodological reasons. The first and most important reason behind them could be that, in this experiment, all information about a character is learned and, therefore, those familiarity effects that certainly play an important role in the formation of these inter-modal interactions should also not work. The second reason could be that this experimental design, using a between-subject format with a rather low number of subjects in each group, probably does not have enough power to detect the small effects that can be observed in the absence of familiarity [29,31]. The stress I place in this commentary on the crucial role played by familiarity factors in the production of inter-sensory communications is, at least in part, because an important contribution to this Special Issue deals just with this question. Stevenage et al. [46] report the results of two experiments that examined face–voice correlations for familiar and unfamiliar stimuli. The authors show that the correlation between facial and vocal processing was significant but small when recognizing unfamiliar individuals, whereas it was much larger when matching familiar subjects. According to the authors, these results support the view that facial and vocal processing are aligned as constituents of an overarching human perception system, but also that familiar and unfamiliar stimuli are processed in different ways. More specifically, the leading role played by familiarity could reflect the importance of a pre-existing mental representation and their cross-talk within the neural architectures when processing familiar faces and voices. In the last paper of this Special Issue to deal with interactions between different channels of human recognition, Zaske et al. [47] consider two problems. The first is that, even if models of inter-sensory integrations specify how visual and auditory information taken from faces and voices is combined, the exact timecourse of this audio-visual face–voice integration remains a matter of debate. The second is that the recognition of people from their voices may be facilitated by ‘voice distinctiveness’, in a manner similar to that which has been reported for faces. Since event-related potentials (ERPs) of an excellent temporal resolution are provided, Zaske et al. [47] use this technique to investigate the timecourse of face–voice-integration, also testing if the recognition of voices may be facilitated by audio-visual learning with distinctive vs. non-distinctive faces. They showed that voices that were previously learned with faces elicited an early (the N250-like) ERP component, which was similar in topography to that typically observed for facial stimuli and that, at the test, voices that were previously learned with distinctive faces were classified faster than those learned with non-distinctive faces. Since the preliminary source localization of the voice-induced N250 was compatible with a source in the fusiform gyrus, these findings provided support to the theory of an early interaction between vocal and facial processing areas during both learning and voice recognition.

## 4. Papers Evaluating the Role That the Voice–Face Connections Can have on the Functional Reorganization in Blind Subjects of the Neural Network for People Recognition

If the integration of vocal and facial signals plays a significant, but not critical role, in facilitating human recognition in subjects who can see, the role that these connections could have for blind subjects, who primarily rely on vocal cues to recognize a person’s identity, is much more relevant. If this role is not investigated, it will remain unclear how the neural systems for voice recognition reorganize themselves in the blind. Two papers have tackled different aspects of this problem in this Special Issue. In the first paper, Pang et al. [48] investigated the alterations in the resting-state functional connectivity among voice- and face-sensitive areas in blind subjects in comparison with controls. These authors found that, in blind subjects, the intranetwork connections among voice-sensitive areas are enhanced, whereas the connections between face- and voice-sensitive areas are diminished. They also suggested that, even if the visual deprivation decreases the internetwork connections between the voice- and face-sensitive areas, these connections may be still involved in the voice recognition processes through subcortical pathways. In particular, they stressed the importance of the connections between the core face-sensitive areas (e.g., FFA and OFA) and the amygdala and of those connecting the amygdala with the inferior frontal gyrus (IFG). Assuming that the face and voice show similar computational processing, and that this helps the functional reorganization [33] of blind individuals and their adaptation to the social environment in daily life, these findings may be consistent with the “metamodal” theory. In the second paper on this topic, Terruzzi et al. [49] tried to integrate neuropsychological and fMRI studies to clarify whether this functional reorganization could be performed by the right temporal lobe. Neuropsychological studies have, indeed, shown that the components of the task of famous voice recognition [20], on which blind subjects obtain their best results, are the same elements selectively impaired in patients with right temporal lesions [50], suggesting that the right temporal lobe may play an important role in this reorganization. The surety of this hypothesis is strengthened using data obtained by Dormal et al. [51] when studying the functional preference for object sounds and voices in the brains of early blind and sighted individuals using fMRI: these authors reported evidence of a selective increase in the functional coupling between the left temporal voice area and the right fusiform gyrus. All these data could, therefore, point to a greater role of the right temporal lobe in the development of the vocal processing abilities of blind subjects than previously understood.

## 5. The Role Played by Familiarity and by Brain Asymmetries in Modality-Specific Recognition Modalities and in the Interactions between These Modalities

We have seen in the previous sections of this Editorial that several studies investigating specific recognition modalities have stressed the role played by familiarity factors in very different conditions, such as the study of Stevenage et al. [43] on voice processing in challenging listening conditions and the research of Chauhan et al. [41] on the relation between the recognition of familiar faces and the identification of emotional facial expressions. We have also reported the results obtained by Stevenage et al. [46] when investigating the role played by familiarity factors in the generation of face–voice interactions. We did so because, in this condition, the size of the face–voice interaction was also much greater when stimuli were familiar than when they concerned unfamiliar subjects. Castro-Laguardia et al. submitted a study specifically devoted to the identification of areas where local fMRI patterns could contribute to familiarity detection of faces, names, or both [52]. These authors identified cortical areas where it was possible to accurately cross-classify familiar stimuli from one category by using a classifier trained with the stimuli from the other ones. They also discovered several areas that supported the classification of familiar faces, but not of familiar names, in the fusiform gyrus, frontal, and temporal regions. They also showed, however, that areas which only contributed to familiarity detection for faces were primarily lateralized to the right hemisphere, and this could be interesting for at least two reasons. The first is that the right lateralization of areas which contributed only to familiarity detection for faces supports the suggestion, advanced in this Special Issue by Terruzzi et al. [49], that the right temporal lobe may play an important role in the development of voice processing abilities of blind subjects. The second is that the right lateralization of areas contributing to the familiarity detection of familiar faces, but not of familiar names, is consistent with the results of clinical studies conducted in patients with unilateral brain lesions and of experimental investigations carried out in healthy subjects. These studies, indeed, showed that (a) facial recognition disorders (prosopagnosia) and vocal recognition disorders (phonagnosia) are more frequent in the right than in left brain-damaged patients [(see [13], and [53] for reviews concerning the psosopagnosia and [54,55] for reviews concerning the phonagnosia) and that (b) these laterality effects can also be found in the recognition of familiar faces, voices, and names by normal subjects (see [14] for review). All these results could have more general implications for the problem of the interactions between different channels of human recognition and the possible underlying mechanisms. Several investigations (e.g., Refs. [29,56]) have, indeed, shown, that an interaction similar to that found between faces and voices is not observed between faces and names, suggesting that channels which process perceptual data are more closely integrated than those which process, respectively, perceptual and verbal data. In previous sections of this Editorial, it has been repeatedly stressed that both the facial and vocal perceptual modalities of person identification are more represented in the right than in the left hemisphere of the brain, whereas the verbal (name) modality is more represented in the left hemisphere. In any case, the existence of an integration between the perceptual modalities subserved by the right hemisphere, but not between those subserved, respectively, by the right and left hemispheres, provides further support to models which assume that an interaction between voice and face sensory systems occurs at the early processing stages, prior to accessing identity nodes.
